# Supplementary material for: Mid-infrared light resonance-enhanced proton conductivity in ceramics
Source: Nat Commun. 2025 Aug 19;16:7707. doi: 10.1038/s41467-025-63027-8 (PMC12365247; doi:10.1038/s41467-025-63027-8)
Supplement: Supplementary file 1 — Supporting Information [file 41467_2025_63027_MOESM1_ESM.pdf]

Supplementary Information

Mid-infrared light resonance-enhanced proton conductivity in ceramics

Haobo Li[1], Yicheng Zhu[1], Zihan Zhao[1], Ruixin Ma[1], Jiachen Lu[1], Wenjie Wan[1,2,], and Qianli Chen[1,]**

1 Global College, Shanghai Jiao Tong University, Shanghai 200240, China

2 School of Physics and Astronomy, Shanghai Jiao Tong University, Shanghai 200240, China

Corresponding authors

Qianli Chen – orcid.org/0000-0001-8460-0596; Email: qianli.chen@sjtu.edu.cn

Wenjie Wan – orcid.org/0000-0002-9743-3480; Email: wenjie.wan@sjtu.edu.cn

Contents

Supplementary Note 1: Characterization of crystal structure, microstructure, and proton concentration and distribution S3

Supplementary Note 2: Effects of atmosphere, proton concentration, and H/D isotopes on proton conductivities S5

Supplementary Note 3: Additional information about the lab-made test system S8

Supplementary Note 4: Additional EIS results S11

Supplementary Note 5: EIS spectra analysis combining equivalent circuit model (ECM) and distribution of relaxation times (DRT) S12

Supplementary Note 6: Model for the grain boundary (GB) conductivity S16

Supplementary Note 7: Thickness-dependent MIR intensity distribution in the samples .. S18

Supplementary Note 8: Impact of IR heating effect on sample temperature S19

Supplementary Note 9: Impact of thermal expansion on sample geometry S20

Supplementary Note 10: Effective potential energy surface (PES) of the proton S22

References S23

Supplementary Note 1: Characterization of crystal structure, microstructure, and proton concentration and distribution

X-ray diffraction (XRD) patterns of protonated and dry $BaZr_{0.8}Y_{0.2}O_{3-\delta}$ (BZY) powder were measured by an X-ray diffractometer (Rigaku Mini Flex 600, Cu Kα, λ = 1.5405 Å). The lattice constants (*a*) for the protonated and dry samples are 4.232 Å and 4.214 Å, respectively, indicating the chemical expansion of lattice due to proton incorporation. O–O separation of protonated BZY was calculated as 2.992 Å by taking $a/\sqrt{(2)}$ for the protonated samples.[1]

SEM images of sintered BZY pellets were characterized by a field emission scanning electron microscope (ThermoFisher Apreo 2 SEM, acceleration voltage 15 kV, magnification 1−2.5 kX). Thermogravimetric analysis (TGA) and differential scanning calorimetry (DSC) were performed on protonated BZY in dry N_2 from room temperature to 900 °C with a heating rate of 10 °C min^{-1} characterized by a simultaneous thermal analyser (STA) (Netzsch STA 449 F3 Jupiter). The proton concentration of protonated BZY was measured by a Karl-Fischer moisture titrator (KEM MKC-710S) in dry N_2 at 900 °C. The Karl-Fischer titration and calculation of proton concentration were conducted following previous work by Han et al.[2]

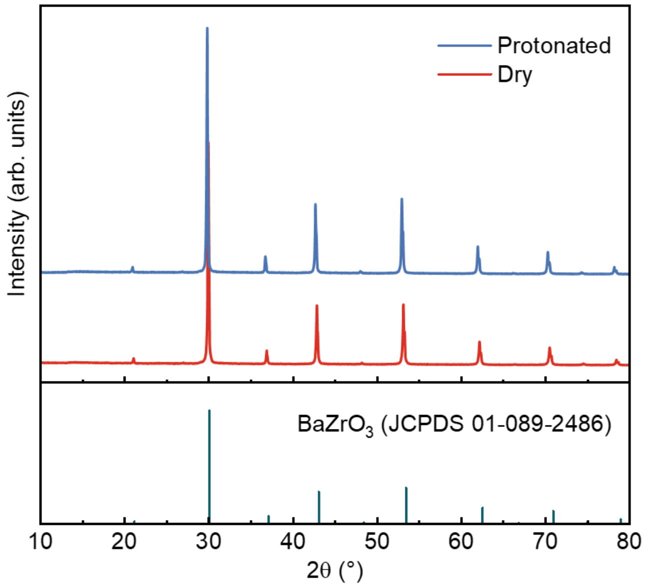

Supplementary Fig. 1. Powder XRD patterns of protonated and dry BZY.

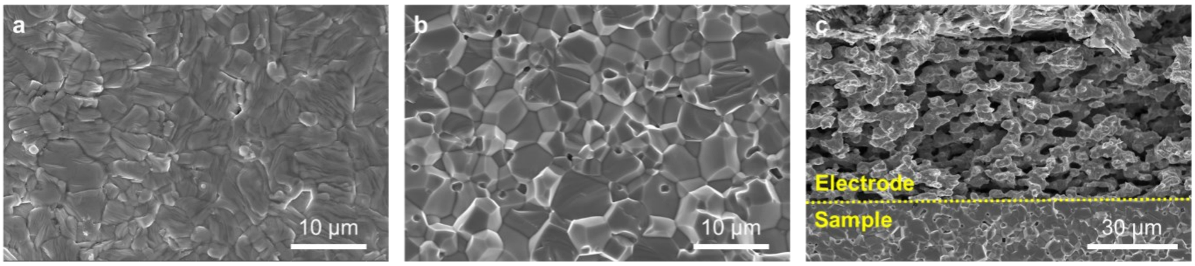

Supplementary Fig. 2. SEM images of **a** surface, **b** cross-section, and **c** electrode-sample interface of the sintered BZY pellets. The pellets are dense with a grain size range of 1−10 μm.

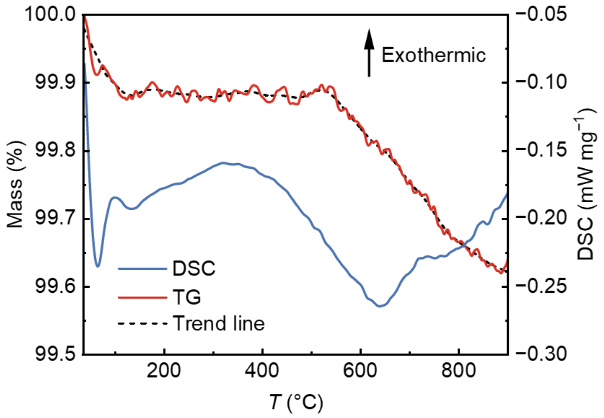

Supplementary Fig. 3. TGA and DSC curves of protonated BZY in dry N_2 from room temperature to 900 °C.

Supplementary Table 1. Proton concentration of protonated BZY measured by a Karl-Fischer moisture titrator in dry N_2 at 900 °C. The errors are the standard deviation for measurements on different samples.

Weight loss (%)	Proton concentration (10^{21} cm^{-3})	Proton content per unit cell	Degree of hydration (%)
0.279±0.006	1.13±0.04	0.0854±0.0019	42.7±1.0

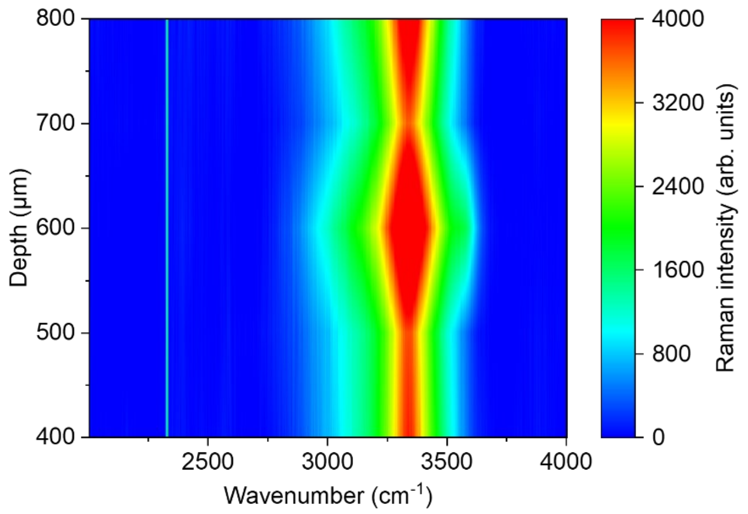

Supplementary Fig. 4. Raman intensity depth profile of O–H stretching band on the cross-section of a protonated BZY pellet of 1.1 mm-thick. A single peak from molecular N_2 in the air appears near 2331 cm^{-1}.[3,4]

Supplementary Note 2: Effects of atmosphere, proton concentration, and H/D isotopes on proton conductivities

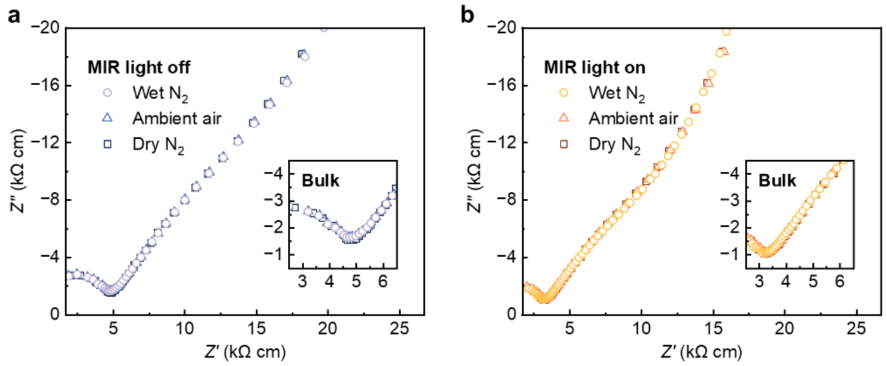

Supplementary Fig. 5. Nyquist plots of protonated BZY samples measured at 160 °C in H_2O-saturated (wet) N_2, ambient air, and dry N_2 with MIR irradiation **a** off and **b** on. The insets illustrate the magnification of bulk features. The atmosphere in the gas-tight chamber was switched from H_2O-saturated N_2 to ambient air, then to dry N_2. The target atmosphere was purged into the chamber for over 1 h at a high gas flow rate before impedance measurements were taken. No significant changes were observed in the impedance spectra in different atmospheres, suggesting no significant hydration/dehydration processes at 160 °C.

Supplementary Table 2. Bulk proton conductivity without MIR irradiation in protonated ($\sigma_{Bulk,wet}$) and dry ($\sigma_{Bulk,dry}$) samples, and proton transport number (t_H) at 160 °C. The protonated and dry samples were prepared separately, as described in Methods. The proton transport number was calculated by $t_H = 1 - \sigma_{Bulk,dry}/\sigma_{Bulk,wet}$.[5] The calculated t_H, close to unity, confirms the dominant proton conduction in BZY20.

Condition	Atmosphere	σ_{Bulk} (S cm^{-1})	t_H
Protonated	Wet N_2	$(2.20\pm0.03)\times10^{-4}$	0.975 ± 0.001
Dry	Dry N_2	$(5.53\pm0.11)\times10^{-6}$	

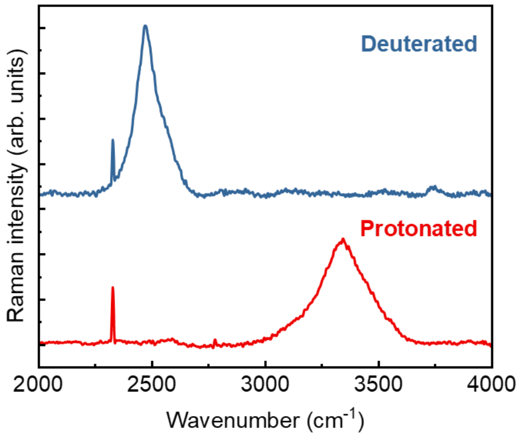

Supplementary Fig. 6. Raman spectra of O–H and O–D stretch vibration bands ($\nu_{O\text{–}H}$ = 3333 cm^{-1}; $\nu_{O\text{–}D}$ = 2472 cm^{-1}) in protonated and deuterated BZY20 samples. The observed results agree with literature.[6] The isotope frequency shift ($\nu_{O\text{–}H}/\nu_{O\text{–}D}$) of 1.347, lower than 1.374 predicted by the harmonic potential model, suggests significant anharmonicity in the potential governing proton transfer.[7,8]

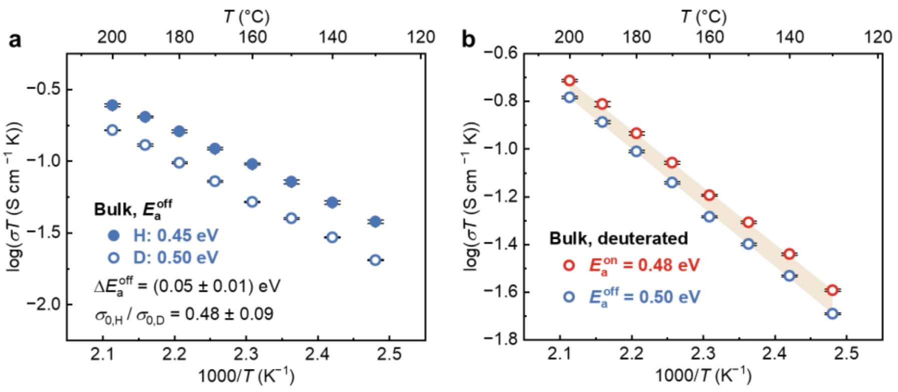

Supplementary Fig. 7. Arrhenius plots of **a** bulk conductivity (σT) of protonated (H, circles) and deuterated (D, open circles) samples measured without MIR irradiation, and **b** deuterated sample measured with (red) and without (blue) MIR irradiation at 130–200 °C. The difference in the activation energies and the ratio of the prefactors of protonated and deuterated samples are marked in **a**, indicating a clear H/D isotope effect.[6]

Supplementary Table 3. Enhancement ratio of protonated samples at $p(H_2O)$ = 0.02 atm, deuterated samples at $p(D_2O)$ = 0.02 atm, and dry samples with $p(H_2O) < 10^{-4}$ atm.

Condition	$(\Delta\sigma/\sigma)_{Bulk}$ (%)	$(\Delta\sigma/\sigma)_{GB}$ (%)
Protonated	36.8±0.51	53.0±6.75
Deuterated	23.1±0.61	33.6±12.2
Dry	3.91±0.14	6.54±0.92

Supplementary Note 3: Additional information about the lab-made test system

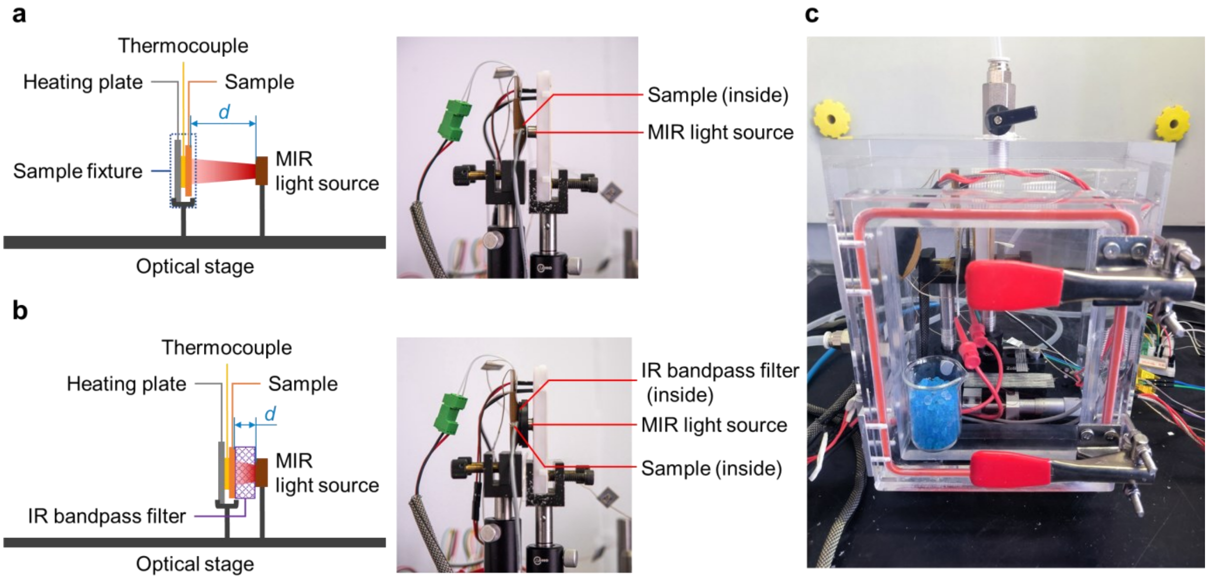

Supplementary Fig. 8. Schematic illustration and optical image for setups **a** without and **b** with the IR bandpass filter of the lab-made test system. *d* denotes working distance (the distance between top surface of the sample and emission window of the light source). The bandpass filter is in contact with sample fixture and the light source. **c** Optical image of the gas-tight chamber enclosing the lab-made test system with internal dimensions of 15 cm × 10 cm × 20 cm. Different atmospheres were obtained by purging the chamber with the target atmospheres for over 1 h.

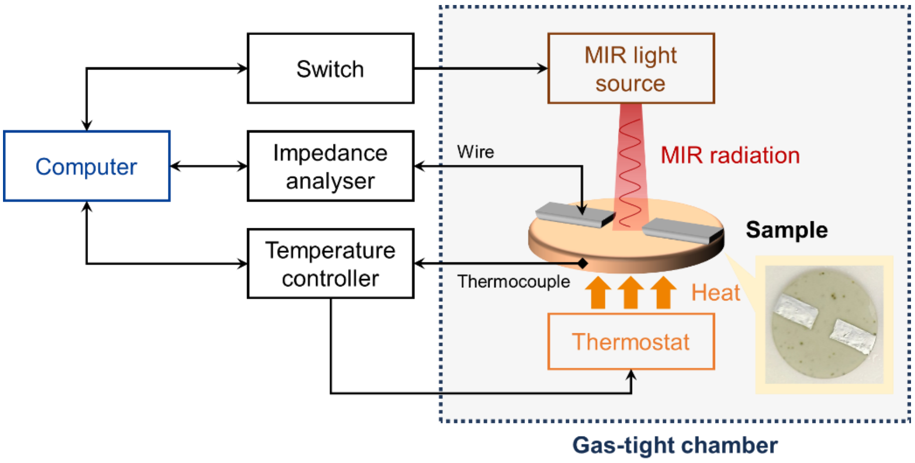

Supplementary Fig. 9. Schematic diagram of the lab-made test system, and optical image of the BZY sample. Black arrows indicate the direction of electrical signals. The feedback loop between the heating plate, sample and temperature controller stabilizes sample temperature.

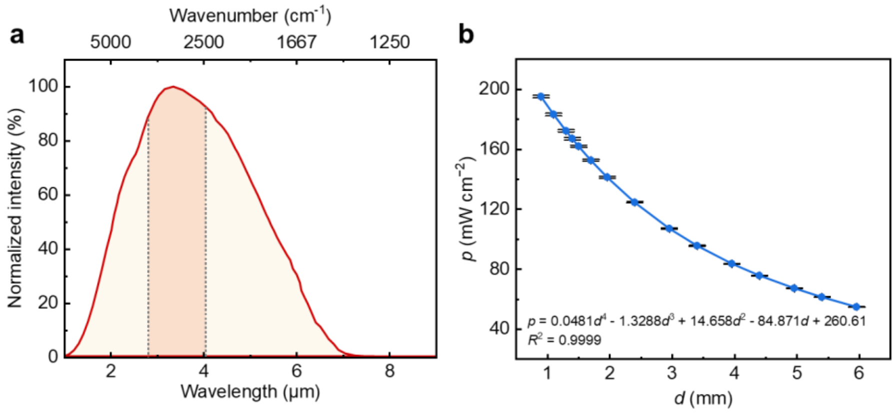

Supplementary Fig. 10. a Radiation spectrum of the MIR light source. The highlighted area (2.7−4.0 μm, orange) corresponds to the major absorption band of O–H stretching vibration and defines the wavelength for the effective power of MIR irradiation (p). **b** p as a function of working distance (d) fitted to a fourth-order polynomial (R^2 = 0.9999). p was estimated from angular radiation distribution of the light source.[9]

Two thermocouples were mounted to the top and bottom surfaces of the pellet to monitor temperature (T), respectively. Both thermocouples showed consistent readings after ~3 min of MIR irradiation (Supplementary Table 4). We chose the bottom thermocouple for T measurements because of better environmental stability (Supplementary Fig. 11), as the top thermocouple was more sensitive to ambient conditions and direct MIR exposure.[10] This

selection is further justified by thermal conduction analysis. The thermal conduction time constant ($\tau_h = C_p h/(\lambda A)$),[11] determined using the sample thickness (h = 0.4 mm), MIR spot area (A = 7 mm²), and measured thermal properties (Netzsch LFA 467) (specific heat capacity C_p = 0.46 J g^{-1} K^{-1}; thermal conductivity λ = 2.1 W m^{-1} K^{-1}), yields $\tau_h \approx 15$ s. This indicates that thermal equilibrium is effectively achieved within $5\tau_h$ (75 s).[11] Our EIS measurements were performed after 3 min of MIR irradiation, ensuring stable temperature distribution in the sample, confirming the appropriateness of our thermocouple configuration.

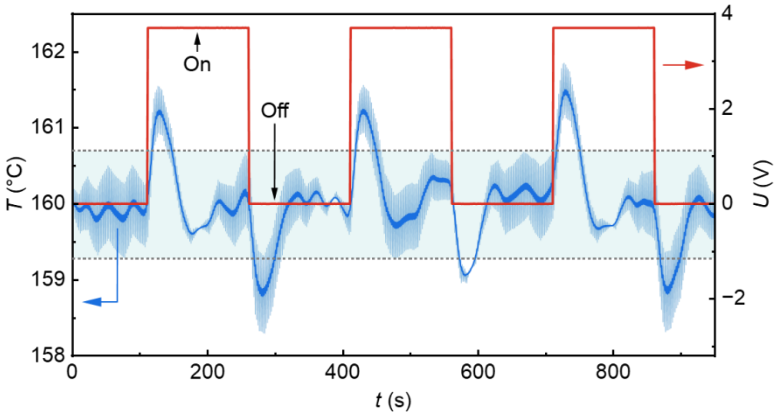

Supplementary Fig. 11. Sample temperature (T) and electrical voltage of the MIR light source (U) during several on/off cycles. Rated voltage of the light source is 3.7 V. Light blue: measured T; blue: 10 s average of T. The fluctuation of T originates from pulse width modulation of the temperature controller regulating output power of the heating plate. The set value of T is 160 °C. T is stabilized at 160 ± 0.7 °C in approximately 100 s after the light source is turned on/off.

Supplementary Table 4. Readings of thermocouples mounted to the sample's top and bottom surfaces after ~3 min of MIR irradiation.

T_{Top} (°C)	T_{Bottom} (°C)
160.8	160.7

Supplementary Note 4: Additional EIS results

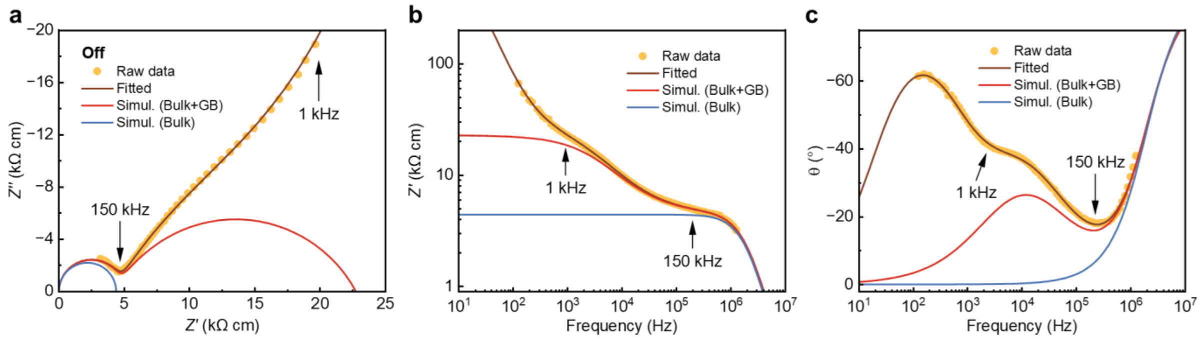

Supplementary Fig. 12. a Nyquist and **b, c** Bode plots of BZY (0.4-mm thick) without MIR irradiation measured at 160 °C in wet N_2. The fitted EIS spectrum, and the deconvoluted bulk and bulk plus GB components of proton conduction are presented. Z' at 150 kHz and 1 kHz reflect bulk and GB conduction features in the EIS spectra, respectively.

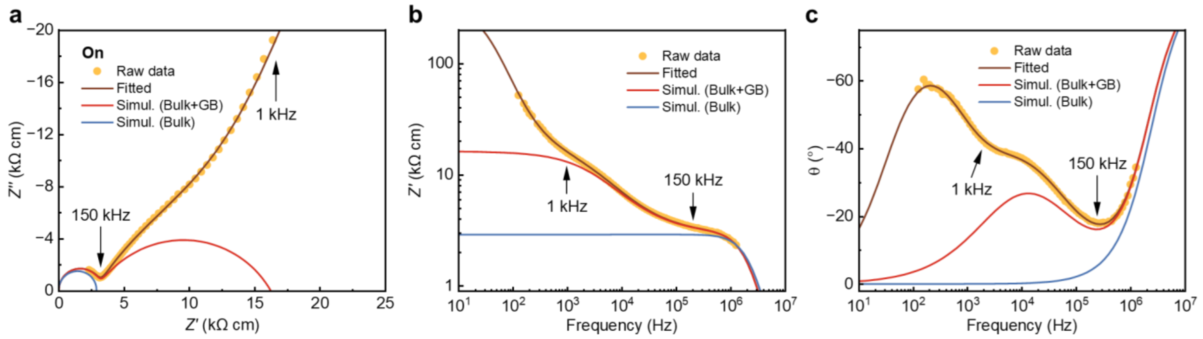

Supplementary Fig. 13. a Nyquist and **b, c** Bode plots of BZY (0.4-mm thick) with MIR irradiation measured at 160 °C in wet N_2. The fitted EIS spectrum, and the deconvoluted bulk and bulk plus GB components of proton conduction are presented. Z' at 150 kHz and 1 kHz reflect bulk and GB conduction features in the EIS spectra, respectively.

Supplementary Table 5. Parameters indicating changes in proton conduction properties under different irradiation states for protonated BZY (0.4-mm thick) measured in wet N_2.

Component	ΔE_a (eV)	$\sigma_0^{on}/\sigma_0^{off}$	Q^{on}/Q^{off}
Bulk	0.041±0.007	0.563±0.073	0.522±0.076
GB	0.018±0.014	1.252±0.190	1.283±0.102

Supplementary Table 6. Parameters indicating changes in proton conduction properties under different irradiation states for protonated BZY (0.9-mm thick) measured in ambient air.

Component	ΔE_a (eV)	$\sigma_0^{on}/\sigma_0^{off}$	Q^{on}/Q^{off}
Bulk	0.017±0.004	0.809±0.029	0.768±0.105
GB	~ 0	1.268±0.140	1.271±0.077

Supplementary Table 7. Enhancement ratios ($\Delta\sigma/\sigma$) of bulk and GB proton conductivity for protonated BZY (0.9-mm thick) measured at 160 °C in ambient air. Considering the small effect of atmosphere (Supplementary Fig. 5), the lower $\Delta\sigma/\sigma$ values are attributed to higher sample thickness (Supplementary Fig. 17).

$(\Delta\sigma/\sigma)_{Bulk}$ (%)	$(\Delta\sigma/\sigma)_{GB}$ (%)
28.6±0.77	41.2±6.95

Supplementary Note 5: EIS spectra analysis combining equivalent circuit model (ECM) and distribution of relaxation times (DRT)

In this work, bulk and GB proton conduction, and electrode polarization processes are not well resolved in the EIS spectra. These overlaps arise when the relaxation times (τ) of the processes differ minimally.[12,13] The fitting errors are considerable (> 60%) while determining the component parameters in such EIS spectra using the equivalent circuit model (ECM) method. On the contrary, the distribution of relaxation times (DRT) method resolves the EIS spectra into continuous curves with distinct peaks, even when the relaxation times of different processes are close.[12,13]

To reduce fitting errors, a combination of ECM and DRT analysis was employed.[14] DRT analysis was performed via DRTtools in MATLAB.[15] Multiple electrochemical processes were identified according to the dominant peaks, and simulated by several R(CPE) elements. The corresponding resistances were estimated by integrating the distribution function over the $\ln\tau$ axis for each peak, and inserted as initial values of different elements in the ECM. In addition,

the errors for all data derived from EIS spectra analysis incorporate standard deviations from three parallel measurements and fitting errors per sample across three samples prepared using the same method.

As presented in Supplementary Fig. 14, three distinct peaks were deconvoluted from the EIS spectra measured at 160 °C, both with and without MIR irradiation. The two peaks covering 10^5–10^7 Hz and 10^3–10^5 Hz were assigned to bulk and GB proton conduction processes, respectively.[13,14,16] The distinct peak below 10^3 Hz, accompanied by several low-intensity, closely spaced hidden peaks, was collectively attributed to electrode polarization and modeled using a single R(CPE) element.[14] Thus, we adopted the equivalent circuit consisting of three serial R(CPE) elements (Supplementary Fig. 14), assuming proton conduction as the origin of the impedance. Each R(CPE) element represents the contribution from the bulk, GB, and electrode to the overall impedance. Supplementary Tables 8–10 show the EIS fitting results, including resistance, capacitance factor, and ideality factor for the bulk, GB, and electrolyte processes. The ideality factors for the bulk and GB processes are close to 1. The changes in capacitance factors and ideality factors for the bulk process upon MIR irradiation are within their errors, suggesting that the changes result from faster bulk transport, but not changes in inductivity. Furthermore, the measured inductance of the wires, as shown in Supplementary Fig. 15, is small and negligible compared to the bulk response, suggesting inductance error[17] contributes negligibly to the EIS measurements.

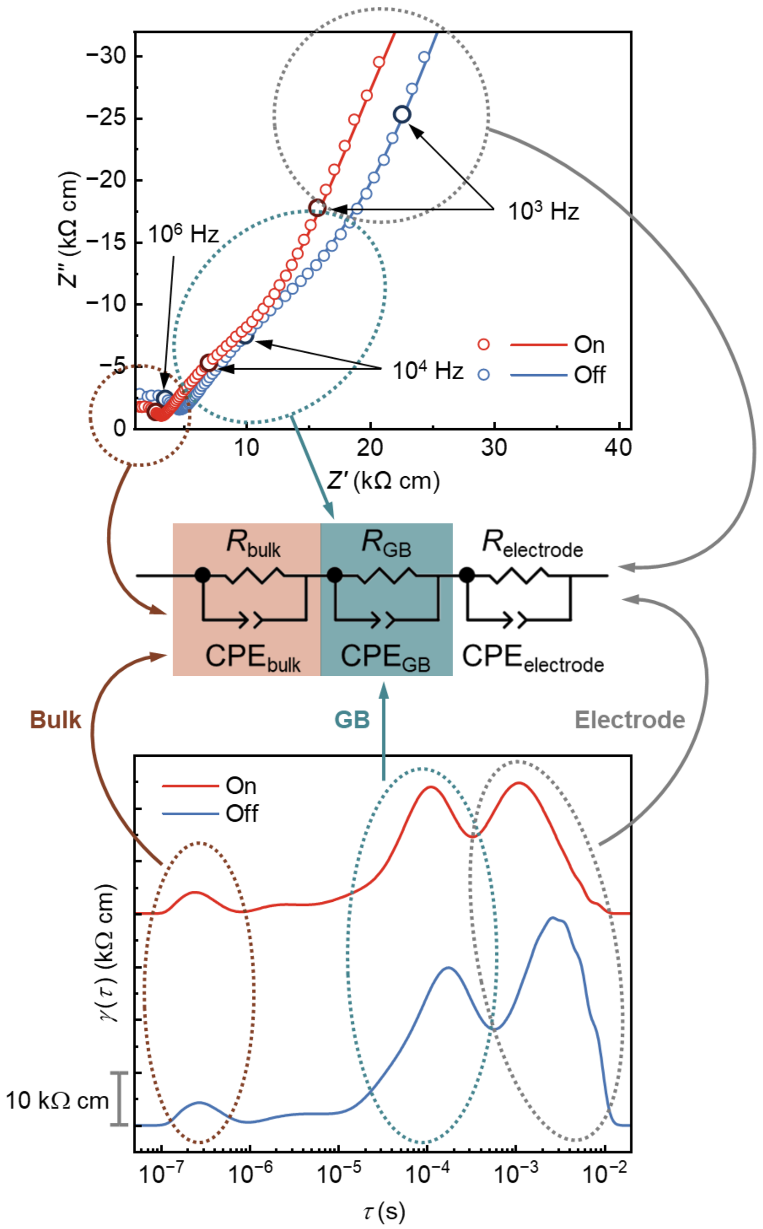

Supplementary Fig. 14. Assignment of different components in EIS spectra (top panel) using the DRT function (bottom panel).[15] The resulting equivalent circuit model (ECM) is presented in the middle panel. The data are measured on the 0.4-mm thick sample at 160 °C in wet N_2.

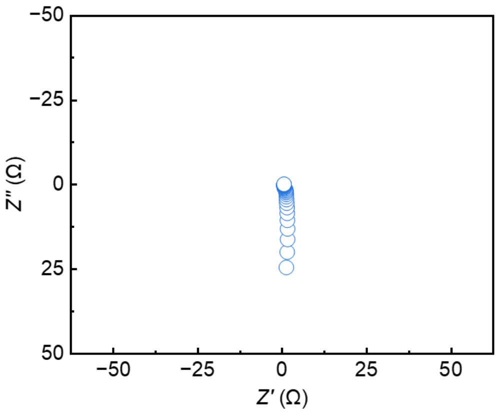

Supplementary Fig. 15. Room-temperature impedance spectrum (2 MHz to 10 Hz) of the test system shorted at the sample position reveals a maximum inductive reactance (positive Z'') originating from the 2-m long leads.[17] This inductive reactance represents less than 0.07% of the total measured Z'' with actual samples at 130–200 °C. Since the leads are kept at room temperature, this result demonstrates that inductance error contributes negligibly to the EIS measurements.

Supplementary Table 8. Fitting parameters of the R(CPE) circuit element for bulk proton conduction: Resistance (R_{Bulk}), capacitance factor (Y_{Bulk}), and ideality factor (n_{Bulk}). Corresponding equivalent circuit model shown in Supplementary Fig. 14.

MIR status	R_{Bulk} (Ω)	Y_{Bulk} (F)	n_{Bulk}
Off	$(1.61\pm0.02)\times10^{5}$	$(2.26\pm0.42)\times10^{-12}$	1.000±0.015
On	$(1.18\pm0.03)\times10^{5}$	$(2.20\pm0.27)\times10^{-12}$	0.999±0.009

Supplementary Table 9. Fitting parameters of the R(CPE) circuit element for GB conduction: Resistance (R_{GB}), capacitance factor (Y_{GB}), and ideality factor (n_{GB}). Corresponding equivalent circuit model shown in Supplementary Fig. 14.

MIR status	R_{GB} (Ω)	Y_{GB} (F)	n_{GB}
Off	$(8.75\pm1.72)\times10^{5}$	$(2.70\pm0.31)\times10^{-10}$	0.912±0.038
On	$(5.72\pm0.93)\times10^{5}$	$(2.52\pm0.49)\times10^{-10}$	0.935±0.031

Supplementary Table 10. Fitting parameters of the R(CPE) circuit element for electrode processes: Resistance ($R_{Electrode}$), capacitance factor ($Y_{Electrode}$), and ideality factor ($n_{Electrode}$). Corresponding equivalent circuit model shown in Supplementary Fig. 14.

MIR status	$R_{Electrode}$ (Ω)	$Y_{Electrode}$ (F)	$n_{Electrode}$
Off	(5.69±0.25)×10^{6}	(5.65±1.09)×10^{-9}	0.545±0.013
On	(3.67±0.07)×10^{6}	(4.23±0.53)×10^{-9}	0.574±0.008

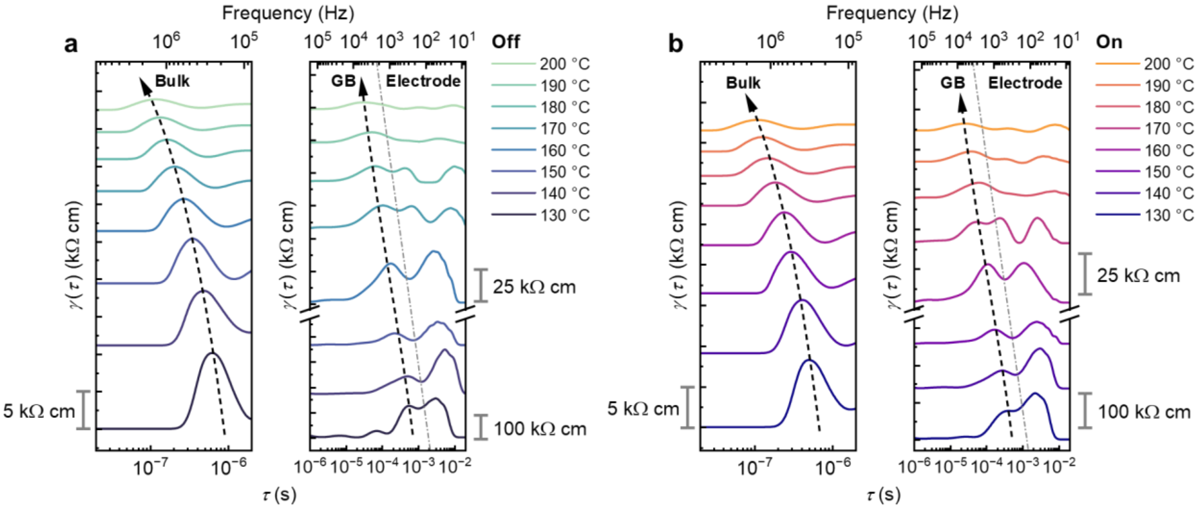

Supplementary Fig. 16. Distribution of relaxation times (DRT) curves[15] for bulk and GB **a** without and **b** with MIR irradiation obtained from the EIS spectra of the samples in 130–200 °C. Considering the inverse relation between the relaxation time and hopping frequency,[14,18] the overall trend of peak position in the DRT spectra well reflects the evolution of jump frequency presented in Figure 3b of the main text.

Supplementary Note 6: Model for the grain boundary (GB) conductivity

The brick-layer model was employed to include the information of microstructure while evaluating grain boundary (GB) conductivity.[19,20] It treats the real microstructure of the samples (Supplementary Fig. 2) as an array of cube-shaped grains separated by flat GB. This model gives two available paths for current conduction: (i) through grains and across GB; (ii) along GB. In BZY20 pellets, proton conduction following path (ii) dominates, where bulk and GB

are connected in series. The specific GB conductivity (termed GB conductivity and σ_{GB} throughout this work) considers the microstructure through the ratio of grain boundary thickness (g) to the grain size (G):[19,21]

$$\sigma_{GB} = \frac{1}{R_{GB}} \frac{d}{A} \frac{g}{G} \tag{S1}$$

where R_{GB} is the apparent GB resistance fitted from EIS spectra, and d and A are the length and cross-sectional area of the sample, respectively. Assuming the dielectric constant is about the same for the bulk and the GB, σ_{GB} can be calculated without microstructure examination: [19,21]

$$\sigma_{GB} = \frac{1}{R_{GB}} \frac{d}{A} \frac{C_{Bulk}}{C_{GB}} \tag{S2}$$

where C_{Bulk} and C_{GB} are the pseudocapacitance of bulk and GB, respectively.

Here, σ_{GB} is approximately two orders of magnitude lower than the bulk conductivity (σ_{Bulk}), primarily due to proton depletion in the space charge layer.[22,23] The GB structure comprises a positively charged core – enriched with oxygen vacancies and protons to accommodate misfit strain – flanked by two negatively charged space charge layers that electrostatically balance the positive charge of GB core.[23] Through the core region, Yang et al.[24] and Bondevik et al.[25] indicated that strong structural distortion and proton segregation at the GB core significantly lower proton mobility. According to the space charge model, the subsequent potential difference between the bulk and the GB core is denoted by φ_B, also known as Schottky barrier height. Kjølseth et al.[23] and Jiang et al.[26] have shown that, when expressed in terms of φ_B, σ_{GB} follows the relation

$$\begin{aligned} \sigma_{GB} &= \sigma_{Bulk} \frac{2e\varphi_B}{k_B T} \exp\left(\frac{-e\varphi_B}{k_B T}\right) \\ &= \sigma_{0,Bulk} \frac{2e\varphi_B}{k_B T^2} \exp\left[\frac{-\left(E_{a,Bulk} + e\varphi_B\right)}{k_B T}\right] \end{aligned} \tag{S3}$$

where σ_{Bulk}, $\sigma_{0,Bulk}$ and $E_{a,Bulk}$ represent bulk proton conductivity, and the corresponding prefactor and activation energy. φ_B can be determined by subtracting the $E_{a,Bulk}$ value from ($E_{a,Bulk} + \varphi_B$) by fitting $\ln(\sigma_{GB} T^2)$ versus 1000 T^{-1}.

As indicated by Eq. (1) in the main text, the on-off ratio of proton conductivity (σ^{on}/σ^{off}) governs the enhancement ratio ($\Delta\sigma/\sigma$). For GB proton conduction, this ratio can be explicitly expressed using Eq. (S3) for a fixed temperature T:

$$\begin{aligned}\frac{\sigma_{\mathrm{GB}}^{\mathrm{on}}}{\sigma_{\mathrm{GB}}^{\mathrm{off}}} &= \frac{\sigma_{0,\mathrm{Bulk}}^{\mathrm{on}}}{\sigma_{0,\mathrm{Bulk}}^{\mathrm{off}}}\frac{\varphi_{\mathrm{B}}^{\mathrm{on}}}{\varphi_{\mathrm{B}}^{\mathrm{off}}}\exp\left[\frac{-\left(\Delta E_{\mathrm{a,Bulk}}+e\Delta\varphi_{\mathrm{B}}\right)}{k_{\mathrm{B}}T}\right] \\ &= \frac{\sigma_{\mathrm{Bulk}}^{\mathrm{on}}}{\sigma_{\mathrm{Bulk}}^{\mathrm{off}}}\frac{\varphi_{\mathrm{B}}^{\mathrm{on}}}{\varphi_{\mathrm{B}}^{\mathrm{off}}}\exp\left(-\frac{e\Delta\varphi_{\mathrm{B}}}{k_{\mathrm{B}}T}\right)\end{aligned} \tag{S4}$$

where $\Delta E_{\mathrm{a,Bulk}} = E_{\mathrm{a,Bulk}}^{\mathrm{on}} - E_{\mathrm{a,Bulk}}^{\mathrm{off}}$ and $\Delta\varphi_{\mathrm{B}} = \varphi_{\mathrm{B}}^{\mathrm{on}} - \varphi_{\mathrm{B}}^{\mathrm{off}}$. When $\varphi_{\mathrm{B}}^{\mathrm{on}} < \varphi_{\mathrm{B}}^{\mathrm{off}}$, both $\varphi_{\mathrm{B}}^{\mathrm{on}}/\varphi_{\mathrm{B}}^{\mathrm{off}} < 1$ and $\Delta\varphi_{\mathrm{B}} < 0$, resulting in a higher on-off ratio and greater enhancement ratio of GB proton conductivity compared to bulk.

For the 0.4-mm thick sample in wet N_2, φ_{B} showed a subtle but consistent decrease from 0.287±0.011 eV (without MIR irradiation) to 0.275±0.009 eV (with MIR irradiation). The lowered φ_{B} may result from mitigated proton segregation at the GB core, as MIR irradiation could enhance proton mobility. This reduction in Schottky barrier height partially explains the greater enhancement ratio of GB proton conductivity with MIR irradiation. By inserting the calculated $\Delta\varphi_{\mathrm{B}}$ and $\varphi_{\mathrm{B}}^{\mathrm{on}}/\varphi_{\mathrm{B}}^{\mathrm{off}}$ values (derived from the φ_{B} analysis) along with $\sigma_{\mathrm{Bulk}}^{\mathrm{on}}/\sigma_{\mathrm{Bulk}}^{\mathrm{off}}$ (obtained from $(\Delta\sigma/\sigma)_{\mathrm{Bulk}}$ at 160 °C in the main text) into Eq. (S4), we obtain $\sigma_{\mathrm{GB}}^{\mathrm{on}}/\sigma_{\mathrm{GB}}^{\mathrm{off}} = 1.72$. This corresponds to $(\Delta\sigma/\sigma)_{\mathrm{GB}} = 72\%$, which is in acceptable agreement with the experimental value (53%) and confirms the model's consistency.

Supplementary Note 7: Thickness-dependent MIR intensity distribution in the samples

To characterize MIR penetration depth, transmitted MIR intensity across protonated samples (0.3–1.1 mm in thickness) was measured using the MIR light source in this work with a HgCdTe (MCT) MIR detector (Healthy Photon HPPD-B-D-04-10; response wavelength 2–4 μm) (Supplementary Fig. 17a). Detector output voltage (u_{MCT}) – proportional to transmitted MIR intensity – was recorded and background-corrected.[27] As shown in Supplementary Fig. 17b, the thickness-dependent attenuation of u_{MCT} follows the Beer-Lambert law, demonstrating measurable MIR penetration through all tested samples.[22] Notably, the 0.4-mm sample exhibited ~1.5× higher transmitted intensity than the 0.9-mm sample.

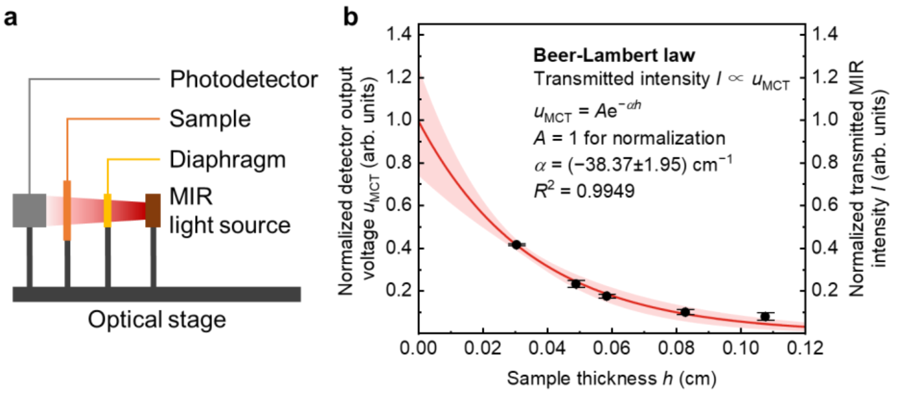

Supplementary Fig. 17. a Schematic illustration for the setup measuring thickness-dependent MIR intensity across the protonated samples. **b** Thickness-dependent attenuation of detector output voltage u_{MCT} (proportional to transmitted MIR intensity I)[27] follows the Beer-Lambert law,[22] demonstrating measurable MIR penetration through all tested samples.

Supplementary Note 8: Impact of IR heating effect on sample temperature

The heating effect of MIR irradiation on the samples is manifested as the percent change in conductivity ($\Delta\sigma/\sigma$) due to the temperature rise (ΔT_{h}) upon irradiation of light. ΔT_{h} is defined by the difference between: (i) the steady-state sample temperature during MIR irradiation while deactivating the PID control, and (ii) the reference temperature T. With the PID feedback loop deactivated, the thermostat's output power was fixed at its baseline level (without MIR irradiation). In this configuration, all observed sample temperature variations can be reasonably attributed to IR heating effect. As plotted in Supplementary Fig. 18, ΔT_{h} was found to be approximately 5 °C when T is 160 °C. The resulting $(\Delta\sigma/\sigma)_{\mathrm{Bulk}}$ and $(\Delta\sigma/\sigma)_{\mathrm{GB}}$ were estimated to be 15% and 25%, respectively, which are lower than those observed in experiment (36.8% and 53.0%). On the other hand, achieving the observed $\Delta\sigma/\sigma$ values solely by heating would require ΔT_{h} = 13 °C. Such contrast demonstrates a higher energy efficiency of MIR irradiation, achieving a comparable enhancement ratio in proton conductivity at significantly lower temperatures.

To quantify ΔT_{h} as a function of working distance (d), we modeled the sample as a thermal resistor considering only conductive heat transfer. We further assumed complete conversion of incident optical power to heat flow (P_{h}), thus having $\Delta T_{\mathrm{h}} \propto P_{\mathrm{h}}$.[22] The thickness-dependent

P_h was calculated by scaling the normalized function $p(d)$ (Supplementary Fig. 10b) to the range [0,1]. As shown in Figures 3c, d of the main text, the observed MIR-induced enhancement in proton conductivity ($\Delta\sigma/\sigma$) for both bulk and grain boundaries exceed the heating-only estimates by a factor of 2–3. This significant discrepancy demonstrates that IR heating effect cannot account for the majority of the enhancement in proton conductivity.

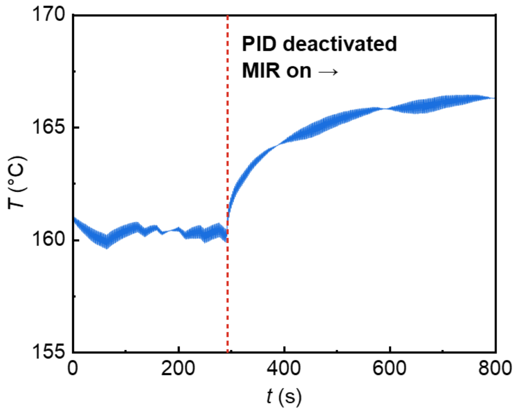

Supplementary Fig. 18. Representative sample temperature curve upon switching on the MIR light source and deactivated PID feedback loop. The temperature rise (ΔT_h) of the sample at equilibrium is approximately 5 °C when the reference temperature is 160 °C. The resulting $(\Delta\sigma/\sigma)_{Bulk}$ and $(\Delta\sigma/\sigma)_{GB}$ were estimated to be 15% and 25%, which are lower than those observed in experiment (36.8% and 53.0%).

Supplementary Note 9: Impact of thermal expansion on sample geometry

In protonic ceramic electrochemical cells (PCECs), thermal expansion coefficient mismatch between electrode and electrolyte materials generates thermal stress under operating conditions. Such stress can initiate microcracks in the cell stack, ultimately leading to significant performance degradation.[28] The impact of thermal expansion with and without MIR irradiation was then evaluated.

To the best of our knowledge, there are no studies reporting material expansion due to optical effects of infrared light. Therefore, we attribute the thermal expansion solely to IR heating effects. The thermal expansion coefficient (α) of BZY20 has been reported to be 8.2×10^{-6} K^{-1}.[29] For an isotropic material with initial volume V_0 at the reference temperature, the thermally

expanded volume due to temperature rise ΔT_h is given by $V_T = V_0\ (1 + \alpha\Delta T_h)^3$, yielding the volume ratio $V_T/V_0 = (1 + \alpha\Delta T_h)^3$.[28]

On the other hand, the proton conductivity σ is expressed as

$$\sigma = \frac{1}{R}\frac{d}{A} = \frac{1}{R}F \quad \text{(S5)}$$

where R, d, and A denote the sample's resistance, length and cross-sectional area, and $F = d/A$ is defined as a geometric factor. By analogy, the geometric factor ratio induced by ΔT_h, $F_T/F_0 = 1/(1 + \alpha\Delta T_h)$ can be obtained.

As discussed in Supplementary Section 8, ΔT_h is approximately 5 °C when the reference temperature is 160 °C. The corresponding changes in V_T/V_0 and F_T/F_0 are both less than 0.001 (Supplementary Fig. 19), confirming that thermal expansion effects are negligible under MIR irradiation. Moreover, 5 °C is lower than the ΔT_h required to achieve the observed $\Delta\sigma/\sigma$ values only through heating (13 °C), implying significantly reduced thermal expansion, and hence thermal stress. These findings validate two key conclusions: (i) Eq. (1) in the main text is valid as the change in sample geometry is within 0.1% during MIR irradiation, and (ii) MIR irradiation would achieve significant proton conductivity enhancement while inducing minimal thermal stress in PCECs – a crucial advantage over conventional heating approach.

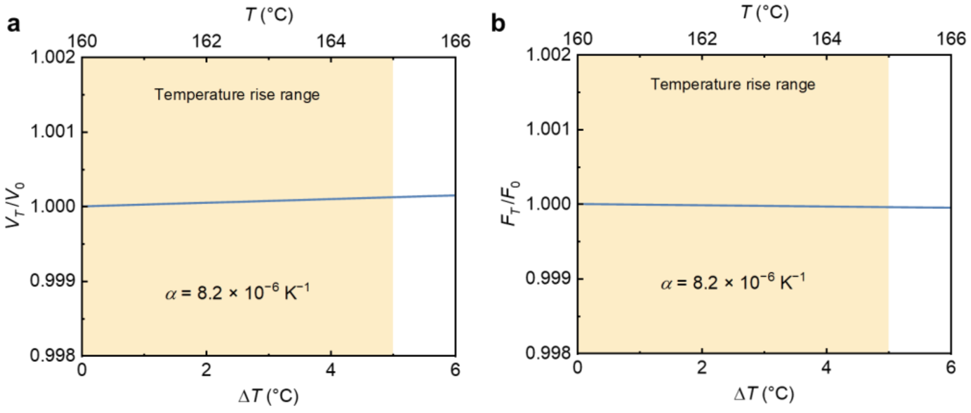

Supplementary Fig. 19. a Volume ratio (V_T/V_0) and **b** geometric factor ratio (F_T/F_0) for calculating proton conductivity showing marginal changes due to IR heating effect.

Supplementary Note 10: Effective potential energy surface (PES) of the proton

As discussed in the main text, the O–H stretching vibration behaves as a highly anharmonic oscillator with a Morse potential V (Eq. (7) in the main text and Supplementary Fig. 20a). The parameter α describes the curvature of the potential and was calculated to be 9.62 $Å^{-1}$ using Python code by Hill.[30] The energy gap between its ground state and the only excited state is 0.41 eV. By absorbing an MIR photon matching its vibration frequency, a small proportion of O–H bonds are brought to their excited state, significantly lowering the effective barrier for proton hopping from 0.46 eV at the ground state to only 0.05 eV. The proportion of such bonds should be positively correlated with the number of MIR photons incident to sample surface, which is proportional to p. Moreover, at the excited state, the O–H stretch amplitude would significantly increase from 0.16 Å to 0.43 Å, as indicated in Supplementary Fig. 20a by the length of the horizontal lines at E_0 and E_1. Thus, at the excited state, the significantly lowered activation barrier and the large O–H stretch amplitude facilitate proton hopping.

A more detailed PES of the proton can be elaborated with a symmetric, effective double-well Morse potential.[31–33] Sakurai et al. suggested a straightforward approach to express the double-well potential by placing two identical Morse potential wells in the opposite direction with a certain separation, using[34]

$$V_{\mathrm{DW}}(r) = V\left(r+\tfrac{R}{2}\right)+V\left(-r+\tfrac{R}{2}\right) \quad \text{(S6)}$$

where R is the nearest O–O separation of protonated BZY (2.992 Å), and V is the Morse potential function. Supplementary Fig. 20b shows the calculated effective double-well potential with the saddle point as the origin of the position coordinate. The distance between two local minima of the potential (l), or the neighbouring sites for proton hopping, is measured as 0.98 Å. Such distance is sufficiently short to facilitate the random diffusion of protons on picosecond timescale ($\tau = 10^{-12}$–10^{-10} s)[31,34,35] in the random walk model ($l^2 = 6D_r\tau$),[36] where the diffusivity D_r of BZY is on the order of 10^{-6}–10^{-4} cm^{-2} s^{-1} between 130 °C and 200 °C in previous works measured by QENS[35,37] and NMR.[1]

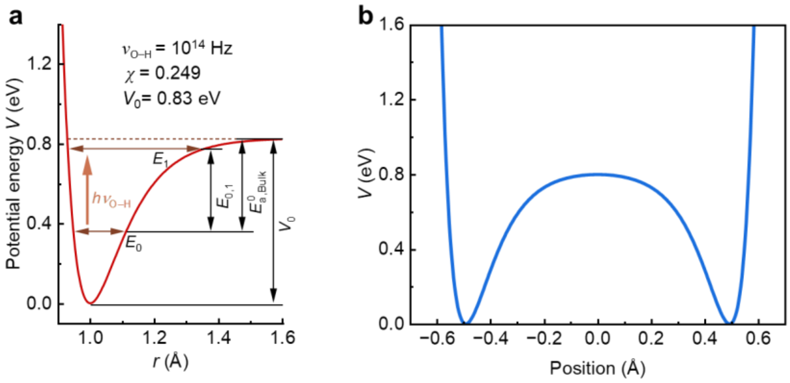

Supplementary Fig. 20. a Effective PES of the proton modelled with the Morse potential where $\nu_{0,1} = 1\times10^{14}$ Hz and $\chi = 0.249$. The vibrational levels and corresponding amplitudes are depicted by the position on the V-axis and the length of the horizontal lines with arrows. The dashed line indicates the top of the activation barrier. **b** Calculated effective double-well Morse potential for the O–H stretching vibration in BZY.

References

1. Yamazaki, Y. *et al.* Proton trapping in yttrium-doped barium zirconate. *Nat. Mater.* **12**, 647–651 (2013).

2. Han, D., Okumura, Y., Nose, Y. & Uda, T. Synthesis of $La_{1-x}Sr_xSc_{1-y}Fe_yO_{3-\delta}$ (LSSF) and measurement of water content in LSSF, LSCF and LSC hydrated in wet artificial air at 300°C. *Solid State Ionics* **181**, 1601–1606 (2010).

3. Lv, H. *et al.* Insertion of N_2 into the Channels of AFI Zeolite under High Pressure. *Sci Rep* **5**, 13234 (2015).

4. Kiefer, J., Stodt, M. F. B. & Fritsching, U. Thermometry by vibrational Raman spectroscopy of nitrogen: Identification and impact of spatial averaging effects. *J Raman Spectroscopy* **52**, 1582–1588 (2021).

5. Saito, K. & Yashima, M. High proton conductivity within the 'Norby gap' by stabilizing a

perovskite with disordered intrinsic oxygen vacancies. *Nat. Commun.* **14**, 7466 (2023).

6. Nowick, A. S. & Vaysleyb, A. V. Isotope effect and proton hopping in high-temperature protonic conductors. *Solid State Ionics* **97**, 17–26 (1997).
7. Varley, J. B., Janotti, A., Singh, A. K. & Van De Walle, C. G. Hydrogen interactions with acceptor impurities in SnO_2: First-principles calculations. *Phys. Rev. B* **79**, 245206 (2009).
8. Bates, J. B. & Perkins, R. A. Infrared spectral properties of hydrogen, deuterium, and tritium in TiO_2. *Phys. Rev. B* **16**, 3713–3722 (1977).
9. INFRASOLID - Thermal infrared emitters. https://www.infrasolid.com/en/product/black-body-infrared-emitters-his550r-0 (2024).
10. Le Maoult, Y. & Schmidt, F. Infrared Radiation Applied to Polymer Processes. in *Heat Transfer in Polymer Composite Materials* (ed. Boyard, N.) 385–423 (Wiley, 2016). doi:10.1002/9781119116288.ch13.
11. Sobota, T. Fourier's Law of Heat Conduction. in *Encyclopedia of Thermal Stresses* (ed. Hetnarski, R. B.) 1769–1778 (Springer Netherlands, Dordrecht, 2014). doi:10.1007/978-94-007-2739-7_384.
12. Lu, Y., Zhao, C.-Z., Huang, J.-Q. & Zhang, Q. The timescale identification decoupling complicated kinetic processes in lithium batteries. *Joule* **6**, 1172–1198 (2022).
13. Dierickx, S., Weber, A. & Ivers-Tiffée, E. How the distribution of relaxation times enhances complex equivalent circuit models for fuel cells. *Electrochimica Acta* **355**, 136764 (2020).
14. Lyagaeva, J. G., Vdovin, G. K. & Medvedev, D. A. Distinguishing Bulk and Grain Boundary Transport of a Proton-Conducting Electrolyte by Combining Equivalent Circuit Scheme and Distribution of Relaxation Times Analyses. *J. Phys. Chem. C* **123**, 21993–

21997 (2019).

15. Wan, T. H., Saccoccio, M., Chen, C. & Ciucci, F. Influence of the Discretization Methods on the Distribution of Relaxation Times Deconvolution: Implementing Radial Basis Functions with DRTtools. *Electrochimica Acta* **184**, 483–499 (2015).
16. Guo, R. & He, T. High-Entropy Perovskite Electrolyte for Protonic Ceramic Fuel Cells Operating below 600 °C. *ACS Materials Lett.* **4**, 1646–1652 (2022).
17. Bonanos, N. *et al.* Applications of Impedance Spectroscopy. in *Impedance Spectroscopy* (eds. Barsoukov, E. & Macdonald, J. R.) 175–478 (Wiley, 2018). doi:10.1002/9781119381860.ch4.
18. Kuzmin, A. V., Plekhanov, M. S. & Lesnichyova, A. S. Influence of impurities on the bulk and grain-boundary conductivity of $CaZrO_3$-based proton-conducting electrolyte: A distribution of relaxation time study. *Electrochimica Acta* **348**, 136327 (2020).
19. Haile, S. M., West, D. L. & Campbell, J. The role of microstructure and processing on the proton conducting properties of gadolinium-doped barium cerate. *J. Mater. Res.* **13**, 1576–1595 (1998).
20. Mariappan, C. R., Yada, C., Rosciano, F. & Roling, B. Correlation between micro-structural properties and ionic conductivity of $Li_{1.5}Al_{0.5}Ge_{1.5}(PO_4)_3$ ceramics. *Journal of Power Sources* **196**, 6456–6464 (2011).
21. Ricote, S., Bonanos, N., Manerbino, A., Sullivan, N. P. & Coors, W. G. Effects of the fabrication process on the grain-boundary resistance in $BaZr_{0.9}Y_{0.1}O_{3-\delta}$. *J. Mater. Chem. A* **2**, 16107–16115 (2014).
22. Defferriere, T., Klotz, D., Gonzalez-Rosillo, J. C., Rupp, J. L. M. & Tuller, H. L. Photo-

enhanced ionic conductivity across grain boundaries in polycrystalline ceramics. *Nat. Mater.* **21**, 438–444 (2022).

23. Kjølseth, C. *et al.* Space–charge theory applied to the grain boundary impedance of proton conducting $BaZr_{0.9}Y_{0.1}O_{3-\delta}$. *Solid State Ionics* **181**, 268–275 (2010).
24. Yang, J.-H., Kim, B.-K. & Kim, Y.-C. Calculation of proton conductivity at the Σ3(111)/[1-10] tilt grain boundary of barium zirconate using density functional theory. *Solid State Ionics* **279**, 60–65 (2015).
25. Bondevik, T., Bjørheim, T. S. & Norby, T. Assessing common approximations in space charge modelling to estimate the proton resistance across grain boundaries in Y-doped $BaZrO_3$. *Phys. Chem. Chem. Phys.* **22**, 11891–11902 (2020).
26. Jiang, L., Norby, T. & Han, D. Thermochemical Expansion and Protonic and Electronic Hole Conductivity of Grain Interior and Grain Boundaries in 10 Mole% Y-Substituted $SrZrO_3$. *ChemSusChem* **16**, e202300661 (2023).
27. HPPD-M-B TEC-cooled HgCdTe (MCT) Amplified Photodetector. https://en.healthyphoton.com/en.healthyphoton.com/upload/file/20200518/20200518185627_11809.pdf (2024).
28. Dayaghi, A. M. *et al.* Increasing the thermal expansion of proton conducting Y-doped $BaZrO_3$ by Sr and Ce substitution. *Solid State Ionics* **359**, 115534 (2021).
29. Lyagaeva, Yu. G., Medvedev, D. A., Demin, A. K., Tsiakaras, P. & Reznitskikh, O. G. Thermal expansion of materials in the barium cerate-zirconate system. *Phys. Solid State* **57**, 285–289 (2015).
30. Hill, C. *Learning Scientific Programming with Python*. (Cambridge University Press, New

York, 2020).

31. Spahr, E. J. *et al.* Giant enhancement of hydrogen transport in rutile TiO_2 at low temperatures. *Phys. Rev. Lett.* **104**, 205901 (2010).

32. Coduri, M., Karlsson, M. & Malavasi, L. Structure–property correlation in oxide-ion and proton conductors for clean energy applications: recent experimental and computational advancements. *J. Mater. Chem. A* **10**, 5082–5110 (2022).

33. Spahr, E. J. *et al.* Proton tunneling: a decay channel of the O–H stretch mode in $KTaO_3$. *Phys. Rev. Lett.* **102**, 075506 (2009).

34. Sakurai, A., Ando, K. & Ashihara, S. Ultrafast proton/deuteron dynamics in $KTaO_3$ observed with infrared pump-probe spectroscopy: Toward understanding of proton conduction mechanism in solid oxides. *J. Chem. Phys.* **149**, 104502 (2018).

35. Braun, A. & Chen, Q. Experimental neutron scattering evidence for proton polaron in hydrated metal oxide proton conductors. *Nat. Commun.* **8**, 15830 (2017).

36. Gao, Y. *et al.* Classical and emerging characterization techniques for investigation of ion transport mechanisms in crystalline fast ionic conductors. *Chem. Rev.* **120**, 5954–6008 (2020).

37. Braun, A. *et al.* Proton diffusivity in the $BaZr_{0.9}Y_{0.1}O_{3-\delta}$ proton conductor. *J. Appl. Electrochem.* **39**, 471–475 (2009).
